# Supplementary material for: Investigation of Nicotianamine and 2′ Deoxymugineic Acid as Enhancers of Iron Bioavailability in Caco-2 Cells
Source: Nutrients. 2019 Jun 30;11(7):1502. doi: 10.3390/nu11071502 (PMC6683067; doi:10.3390/nu11071502)
Supplement: Supplementary file 1 [file nutrients-11-01502-s001.pdf]

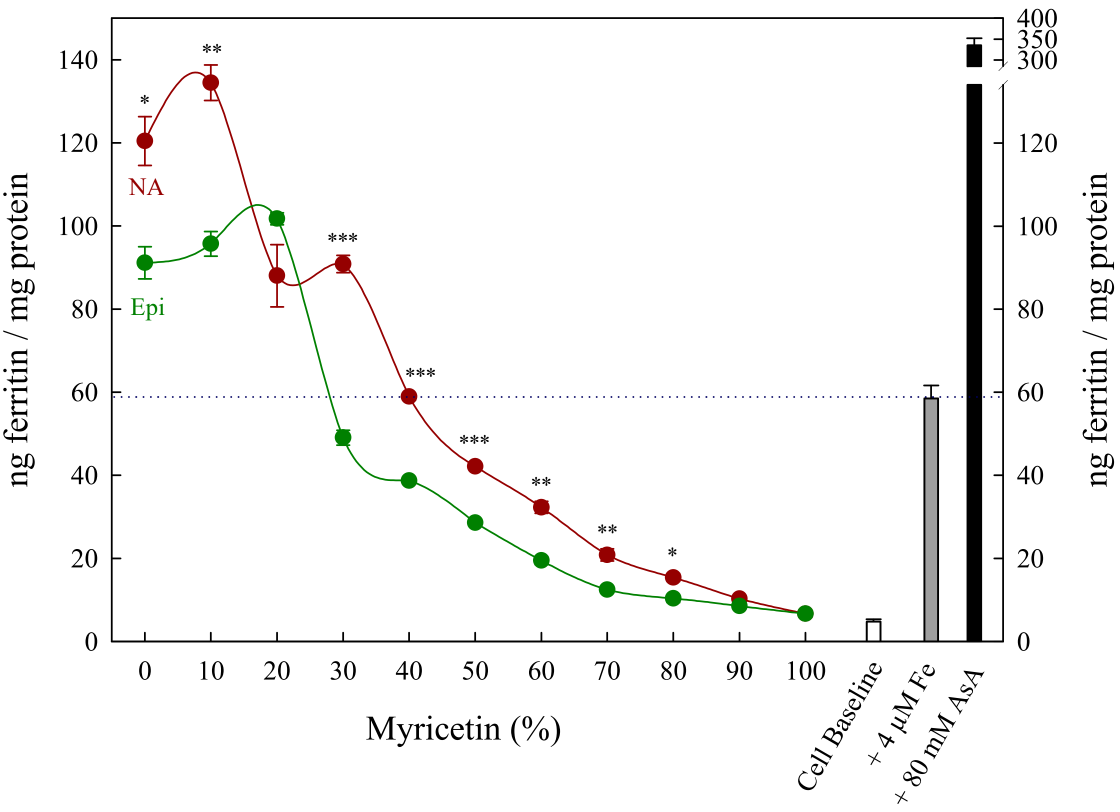

Figure S1 Ferritin formation in Caco-2 cells in response to various molar ratios of nicotianamine : myricetin (NA, red) and epicatechin : myricetin (Epi, green). Total metabolite concentration at each data point was 10 μM. Dotted line indicates ferritin response to 4 μM Fe alone and is extended to both y axes to facilitate comparison with other treatments. Error bars represent standard error of the mean of three replicates. Asterisks denote the significance between NA and Epi at each molar ratio for $p < 0.05$ (*), $p \leq 0.01$ (**), $p \leq 0.001$ (***) as determined by Student's t-test.

Table S1 Fold increases in Caco-2 cell ferritin formation in response to different molar ratios of 2′ deoxymugineic acid (DMA, blue), epicatechin (Epi, green), ascorbic acid (AsA, orange), and nicotianamine (NA, red) compared to ferritin formation in the presence of Fe alone. For each metabolite the color intensity indicates peak fold increase across the range of molar ratios to Fe.

Molar Ratio to Fe	DMA	Epi	AsA	NA
0.05	1.13	1.16	1.29	1.35
0.1	1.48	1.26	1.45	1.51
0.125	-	1.15	1.23	1.22
0.2	1.44	1.5	1.63	1.78
0.25	-	1.31	1.67	1.82
0.3	2	1.62	1.52	2.24
0.4	2.08	1.57	1.77	2.18
0.5	1.94	1.64	1.72	2.43
1	0.68	1.51	2.09	2.66
1.5	-	2.25	2.01	5.03
2	0.63	2.06	2.66	4.05
3	0.64	1.58	-	1.54
4	0.68	1.69	-	1.51
5	0.44	1.48	-	1.28
20	-	-	8.08	-
